# Anti-Inflammatory and Gut Microbiota Modulating Effects of Probiotic *Lactobacillus paracasei* MSMC39-1 on Dextran Sulfate Sodium-Induced Colitis in Rats

**DOI:** 10.3390/nu15061388

**Published:** 2023-03-13

**Authors:** Boonyarut Ladda, Chittapon Jantararussamee, Wisuit Pradidarcheep, Anongnard Kasorn, Udomlak Matsathit, Malai Taweechotipatr

**Affiliations:** 1 Center of Excellence in Probiotics, Srinakharinwirot University, Bangkok 10110, Thailand; 2 Department of Microbiology, Faculty of Medicine, Srinakharinwirot University, Bangkok 10110, Thailand; 3 Department of Anatomy, Faculty of Medicine, Srinakharinwirot University, Bangkok 10110, Thailand; 4 Department of Basic Medical Science, Faculty of Medicine Vajira Hospital, Navamindradhiraj University, Bangkok 10300, Thailand; 5 Department of Food Science and Nutrition, Faculty of Science and Technology, Prince of Songkla University, Pattani 94000, Thailand

**Keywords:** probiotic, *Lactobacillus*, colitis, tumor necrosis factor-α, dextran sulfate sodium

## Abstract

Probiotics have been shown to possess several properties, depending on the strain. Some probiotics have important roles in preventing infection and balancing the immune system due to the interaction between the intestinal mucosa and cells in the immune system. This study aimed to examine the properties of three probiotic strains using the tumor necrosis factor-alpha (TNF-α) inhibition test in colorectal adenocarcinoma cells (Caco-2 cells). It was revealed that the viable cells and heat-killed cells of the probiotic *L*. *paracasei* strain MSMC39-1 dramatically suppressed TNF-α secretion in Caco-2 cells. The strongest strains were then chosen to treat rats with colitis induced by dextran sulfate sodium (DSS). Viable cells of the probiotic *L*. *paracasei* strain MSMC39-1 reduced aspartate transaminase and alanine transaminase in the serum and significantly inhibited TNF-α secretion in the colon and liver tissues. Treatment with the probiotic *L*. *paracasei* strain MSMC39-1 alleviated the colon and liver histopathology in DSS-induced colitis rats. Furthermore, supplementation with probiotic *L*. *paracasei* strain MSMC39-1 increased the genus *Lactobacillus* and boosted the other beneficial bacteria in the gut. Thus, the probiotic *L*. *paracasei* strain MSMC39-1 exhibited an anti-inflammation effect in the colon and modulated the gut microbiota.

## 1. Introduction

Probiotics are live microorganisms with several properties, such as protection of the gut epithelium barrier, pathogen inhibition and immunomodulation. The properties of probiotics are strain-dependent. *Lactobacillus*, *Bifidobacteria*, *Enterococcus*, *Streptococcus*, *Bacillus*, *Lactococcus* and *Saccharomyces* are the common probiotic strains used [1]. Probiotics can be isolated from various sources including local fermented food, raw milk, yogurt, infant feces and flowers. These microorganisms are beneficial microbiota which alleviate human diseases including inflammatory bowel disease (IBD), obesity, alcoholic liver disease and allergies [2].

Ulcerative colitis (UC) [3] is a type of IBD that causes inflammation and irritation in the digestive system. In IBD patients, levels of pro-inflammatory cytokines including tumor necrosis factor-alpha (TNF-α), interleukin-1 (IL-1) and interleukin-17 (IL-17) are increased [4]. TNF-α seems to be the potent acting mediator in IBD pathogenesis [5]. The main symptoms of UC are frequently diarrhea, weight loss and bloody stool [3]. The choices for treatment of UC include the use of anti-TNF, antibiotics, the increment of good microbiota by fecal transplantation or surgery [3].

Gut microbiota is the microorganism found ubiquitously in the gastrointestinal tract. This microbiota benefits its host in digestion, detoxification, production of anti-microbial substance and balancing the immune system [6]. However, dysbiosis or an imbalance of microbiota in the composition and abundance of different bacterial commensal communities can occur. In this condition, the pathogenic microbes outnumber the beneficial microbiota, leading to the destruction of the intestinal barrier and the activation of TNF-α production, resulting in intestinal inflammation and IBD pathogenesis [7]. There are several in vitro and in vivo studies on the properties of probiotics using colitis models. The *L*. *plantarum* strain reduced TNF-α, interleukin-6 (IL-6) and interleukin-8 (IL-8) cytokine secretion in lipopolysaccharide-stimulated Caco-2 human intestinal epithelial-like cells [8]. A recent study reported that heat-treated encapsulated *L*. *kefiranofaciens* improved colon inflammation in 2% dextran sodium sulfate (DSS) colitis mice [9]. In addition, live probiotic *L*. *johnsonii* suppressed TNF and IL-1β expression and stimulated anti-inflammatory cytokine (IL-10) expression in the colon of trinitrobenzenesulfonic acid (TNBS)-induced colitis mice [10]. Furthermore, previous studies revealed that oral gavage of live *L*. *sakei* alleviated symptoms and histopathology of colitis and reduced the expressions of pro-inflammatory cytokines (TNF-α, IL-1 and IL-6) in the colons of DSS-induced colitis animals [11]. In vitro and in vivo studies showed that *L*. *plantarum* LS/07 activated IL-10 production in macrophages derived from blood monocytes and improved weight loss and diarrhea signs of colon inflammation in dextran sulphate sodium (DSS)-induced colitis rat models [12]. *L*. *paracasei* subsp. *paracasei* NTU 101 could also reduce TNF-α and IL-6 production in colon tissue, lower oxidative stress and increase *Bifidobacterium* spp. in the feces and the gastrointestinal tract of DSS-induced colitis mice [13]. A recent study showed that *Bifidobacterium animalis* subsp. *lactis* strain BB12 inhibited TNF-α production and reduced apoptosis in the intestinal epithelium [14]. Oral gavage with encapsulated *L*. *rhamnosus* strain GG lowered colon inflammation by inhibiting TNF-α production [15]. Crude *Gleditsia sinensis* Lam. extract combined with *L*. *brevis* inhibited TNF-α, IL-1β and IFN-γ production in the lymph nodes and spleens of 4% DSS-induced colitis mice [16]. Oral gavage with *L*. *curvatus* isolated from cabbage reduced colitis pathogenesis and induced IL-10 production in bone marrow-derived dendritic cells (BMDCs) [17]. Combined administration of *L*. *casei* and *Pistacia atlantica* reduced colitis and myeloperoxidase, an enzyme for the detection of neutrophils in colon inflammation, in 2, 4, 6-Trinitrobenzene sulfonic acid (TNBS)-induced ulcerative colitis rats [18]. Administration of *Saccharomyces cerevisiae* and *L*. *plantarum* in rice bran and *Lactococcus lactis* improved colon inflammation in DSS-induced mice [19]. Red ginseng fermented with the probiotic *L*. *plantarum* decreased IL-6 and TNF-α in the serum and colon tissue and decreased the severity of colitis in DSS-induced colitis mice [20]. Probiotics *Enterococcus faecium*, *L*. *helveticus* and *B*. *longum* in soy decreased the severity of colitis and increased *Lactobacillus* spp. and *Bifidobacterium* spp. in DSS-induced colitis rats [21]. *L*. *fermentum* reduced the signs of colitis and pro-inflammatory cytokines such as TNF-α, IL-6, IFN-γ and IL-12 [22]. *L*. *paracasei* alleviated intestinal inflammation by reducing the number of neutrophils and pro-inflammatory cytokines (IL-1β and IL-6) [23]. Synbiotic between *L*. *paracasei* and prebiotics reduced colon damage and neutrophils and protected the intestinal epithelial barrier from damage in colitis mice [24]. However, there are no studies regarding the anti-inflammatory and gut microbiota modulating effects of probiotic strains isolated from healthy infant feces, which were used in the present study. In our previous studies, this strain of probiotics exhibited significant potential in inhibiting TNF-α production in THP-1 cells [25], reducing liver fibrosis [26], improving alcoholic hepatitis [27] and lower gingival pain [28] and curing acne vulgaris [29]. This study, therefore, aimed to investigate the anti-inflammatory and gut microbiota modulating effects of the probiotic *L*. *paracasei* MSMC39-1 in DSS-induced colitis rat.

## 2. Materials and Methods

### 2.1. Probiotic Culture Supernatant and Viable Cells Preparation

Three probiotic strains, including *L*. *paracasei* MSMC 39-1, *L*. *casei* MSMC39-3 and *Weissella confusa* strain MSMC57-1, were cultivated on de Man–Rogosa–Sharpe (MRS) agar (Himedia, Mumbai, India) and incubated in anaerobic conditions for 48 h at 37 °C. The final concentration of 10^9^ CFU/mL of probiotic strain culture supernatant was separated using centrifugation and filtration with 0.22 μm and was intensified using a vacuum centrifuge concentrator (Christ, Osterode am Harz, Germany) for 4–6 h at 40 °C [25]. Concentrated probiotic strains were resuspended in serum-free medium DMEM and stored at −20 °C until use. Viable cells were prepared by adjusting the final concentration of probiotic strains to 10^9^ CFU/mL [25] and washing two times with 1xPBS. Probiotic strains were re-suspended with PBS and used instantly for TNF-α production in Caco-2 cell line.

### 2.2. Heat-Killed Cells Preparation

The probiotic strains were cultivated in MRS broth, and incubated anaerobically at 37 °C for 24–48 h. The final concentration of 10^9^ CFU/mL of probiotic strains was centrifuged and washed with 1× PBS. The pellets of probiotic strains were then re-suspended in 1x PBS, boiled for 1 h at 85 °C and stored at −20 °C. Cell survival was re-checked using T-streaking on MRS medium. The cells were then incubated anaerobically for 24–48 h at 37 °C [25].

### 2.3. Sonicated Cells Preparation

The strains were cultivated on MRS medium and anaerobically incubated for 48 h at 37 °C. The pure colonies of each strain were cultivated in MRS broth and incubated in anaerobic condition at 37 °C for 24–48 h. The final concentration of 10^9^ CFU/mL [25] of the strains was centrifuged and resuspended in serum-free Dulbecco’s Modified Eagle Medium (DMEM). Sonication of the strains was set at speed 85–88% for 7.10 min by using ultrasonic homogenizers (Sonopuls, Bandelin, Germany), and the strains were stored at −20 °C.

### 2.4. Caco-2 Cells

Caco-2 colorectal adenocarcinoma cells (ATCC, HTB 37) were purchased from the American Type Culture Collection (ATCC). The Caco-2 cells were cultured in high glucose DMEM (Gibco-invitrogen, Carlsbad, CA, USA) and supplemented with 10% fetal bovine serum (FBS), 1% penicillin (100 units/mL) and streptomycin (100 mg/mL) and incubated at 37 °C in a 5% CO_2_ incubator, 98% humidified.

### 2.5. The effect of Probiotic Strains in the TNF-α Production in Caco-2 Cells

Caco-2 cells at 5 × 10^5^ cells/mL were seeded on a six-well plate in high glucose DMEM supplemented with 10% FBS and 1% penicillin-streptomycin in a 37 °C incubator, 98% humidified and at 5% CO_2_. After 15 days, the plate was washed in 1× PBS and replaced with 1% FBS antibiotic-free DMEM [8]. Each well was treated with 10% condition media (CM), 20% sonicated cells (SON) and the heat-killed or viable cells of three probiotic strains and activated with 20 ug/mL lipopolysaccharide from *Escherichia coli* (Sigma, Saint Louis, MO, USA) for 24 h. Supernatants were collected by centrifugation at 3000 rpm, 4 °C for 5 min. Levels of TNF-α production were measured in the supernatants via a sandwich ELISA kit (R&D Systems, Minneapolis, MN, USA). Experiments were performed with duplicated samples.

### 2.6. Animal Experiment

For the colitis model, 24 male Wistar rats (*Rattus norvegicus*) aged between 8 and 10 weeks and weighing 250–300 g were obtained from Namura-Siam International (Bangkok, Thailand). The animal housing was set at a temperature of 22 ± 5 °C, humidity of 55 ± 5% and 12 h: 12 h light/dark at the Medical Center Animal Care Laboratory, Srinakharinwirot University, Thailand. The rats were acclimatized to the environment for 1 week before starting the experiment. All male rats were selected for this experiment to avoid a sex hormone effect. The animal protocol and experiments were conducted under the license no. COA/AE-002-2563 from the Animal Ethics Committee of the Srinakharinwirot University.

### 2.7. Induction of Colitis

Colitis rats were induced by mixing 3 % w/v Dextran sulfate sodium (DSS) (molecular weight approx. 40 kDa; TCI, Tokyo, Japan) into their drinking water for 2 weeks. All animals were fed with a standard diet ad libitum [30].

### 2.8. Experimental Designs

**Figure 1:**
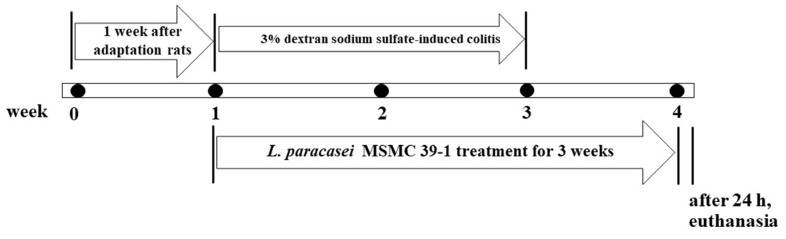
Timeline of the dextran sodium sulfate-induced colitis rat model. The 3% dextran sodium sulfate was administered to each rat for 2 weeks. MSMC39-1 was administered for 3 weeks, *n* = 6 rats.

*L*. *paracasei* strain MSMC39-1, the strongest strain in the TNF-α inhibition test in Caco-2 cells, was selected for animal experiments. Rats were divided into four groups (*n* = 6 for each group). The normal control group (group 1) received a standard diet and drinking water. The probiotic control group (group 2) was administrated with viable cells of probiotic *L*. *paracasei* strain MSMC39-1 by oral gavage and fed a standard diet. The colitis rats (group 3) received a standard diet with 3% DSS in their drinking water for 2 weeks. The probiotic-treated rats (group 4) obtained a standard diet with 3% DSS in their drinking water for 2 weeks and were then administered with probiotic *L*. *paracasei* strain MSMC39-1 for 3 weeks. The same probiotics were administered in the probiotic control and probiotic-treated groups. Rats were orally gavaged with viable probiotic *L*. *paracasei* MSMC39-1 at 1 × 10^9^ CFU/mL/rat/day. Rats were weighed daily in gram units. The timeline of the experiment is shown in Figure 1.

### 2.9. Detection of Aspartate Aminotransferase and Alanine Aminotransferase

After all rats were anesthetized with isoflurane (Attane, Bethlehem, PA, USA), blood was collected from a cardiac puncture. Serum was collected by centrifuging the blood sample at 3500 rpm for 10 min. The levels of aspartate aminotransferase (AST) [31] and alanine aminotransferase (ALT) [32] were measured by a standard clinical lab (Professional Laboratory Management Corp company, Bangkok, Thailand).

### 2.10. Detection of TNF-α in the Colon and Hepatic Tissue

After 24 h of oral gavage with *L*. *paracasei* at the end of the experiment, the body weight of the rats were measured immediately. The rats were then euthanized under anesthesia with isoflurane. The large intestine and liver were removed, weighed, rinsed and excised. Colon and liver tissues were homogenized with radioimmunoprecipitation assay (RIPA) lysis buffer and centrifuged. The supernatants were collected for TNF-α detection using an ELISA kit (R&D Systems, Minneapolis, MN, USA). The colon lengths were measured in centimeter units.

### 2.11. Histology Evaluation of Colitis Rats

Colon and liver tissues were fixed in 4% paraformaldehyde at 4 °C for histopathology evaluation [33]. The specimens were dehydrated in ethanol series, embedded in paraffin blocks and sectioned at 5–7 μM. The sectioned specimens were deparaffinized with xylene, rehydrated with ethanol series and stained with hematoxylin and eosin (H&E). The slides were dehydrated in ethanol series, cleared in xylene and mounted with Permount^®^. The histopathology examination of colitis rats was performed with a light microscope (Olympus UC50, Tokyo, Japan) at ×20 and ×40 magnifications, and the measurements were scored [33] as shown in Table 1.

### 2.12. Microbiota Detection using 16S rDNA Next-Generation Sequencing (NGS)

At the end of the probiotic-treated colitis experiment, rat feces were collected for the detection of microbiota modulation [24] using 16S rDNA NGS. Stool DNA extraction was carried out using a QIAamp Fast DNA Stool Mini Kit (Qiagen, Hilden, Germany). Stool DNA concentration was detected using a nanodrop spectrophotometer (Thermo Scientific, Waltham, MA, USA). The two primers used were as follows: forward primer: 5′TCGTCGGCAGCGTCAGATGTGTATAAGAGACAGCCTACGGGNGGCWGCAG3′ and reverse primer: 5′GTCTCGTGGGCTCGGAGAGTGTATAAGAGACAGGACTACHVGGGTATCTAAT CC3′. Two variable regions of 16S rDNA (V3 and V4) were sequenced using Illumina Miseq sequencing (Illumina, San Diego, CA, USA).

### 2.13. Statistical Analysis

The statistical significance of differences was assessed using GraphPad Prism version 8.00. One-tailed Student’s t-test was used in the analysis of the in vitro study. A *p*-value ≤ 0.05 was considered to be statistically significant. One-way analysis of variance (ANOVA) was used in the analysis of the in vivo study to make comparisons with the control group, and the findings were expressed as mean ± SD.

## 3. Results

### 3.1. The effect of Probiotic Strains on TNF-α Production in Caco-2 Cells

Previous research exhibited that *L*. *paracasei* strain MSMC 39-1, *L*. *casei* strain MSMC 39-3 and *W. confusa* strain MSMC57-1 suppressed TNF-α secretion in the THP-1 monocytic cell line [25]. In this study, the effects of *L*. *paracasei* MSMC39-1, *L*. *casei* MSMC39-3 and *W. confusa* MSMC57-1 on TNF-α production were examined in Caco-2 cells stimulated with lipopolysaccharides.

**Figure 2:**
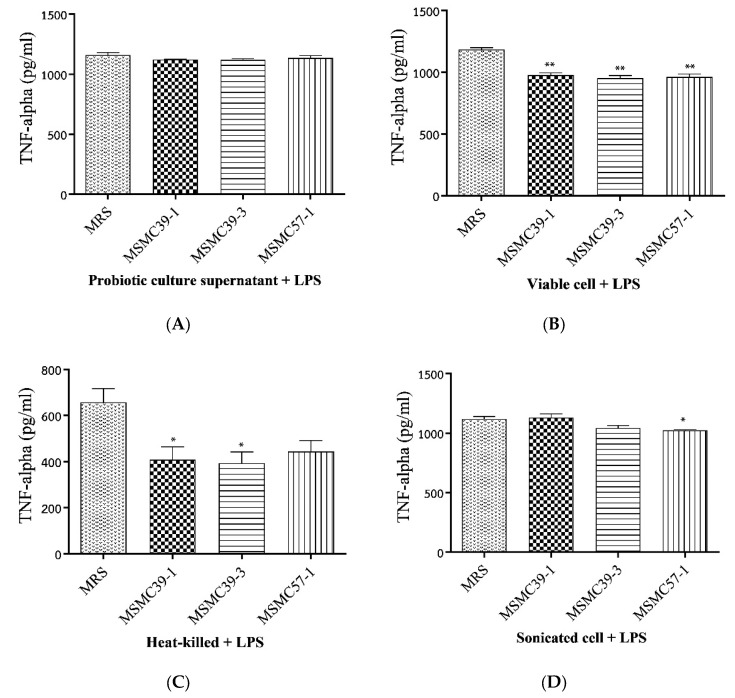
Effects of probiotic strain MSMC39-1, strain MSMC39-3 and strain MSMC57-1 ((**A**) Probiotic culture supernatant, (**B**) Viable cells, (**C**) Heat-killed cells and (**D**) Sonicated cells on the TNF-α secretion in Caco-2 cells stimulated with lipopolysaccharides. Experiments were performed in duplicate with MRS control. One-tailed Student’s t-test was used for statistical analysis. Error bars show standard deviations (SD), * (*p* < 0.05) and ** (*p* < 0.01).

Four antigens of strain MSMC39-1, strain MSMC39-3 and strain MSMC57-1, including probiotic culture supernatant, viable cells, heat-killed cells and sonicated cells, were incubated with Caco-2 cells stimulated with LPS for 24 h prior to evaluation of TNF-α levels. The results showed that probiotic culture supernatants of MSMC 39-1, MSMC 39-3 and strain MSMC57-1 had no influence on TNF-α production in the Caco-2 cell line, as shown in Figure 2A. Strong inhibitions of TNF-α production were observed when viable cells of strain MSMC 39-1, strain MSMC 39-3 and strain MSMC57-1 were incubated with Caco-2 cells, as shown in Figure 2B (** *p* < 0.01). TNF-α secretions in Caco-2 cells were significantly decreased by the addition of heat-killed cells of MSMC 39-1 and MSMC 39-3, as shown in Figure 2C (* *p* < 0.05). In addition, heat-killed cells of strain MSMC57-1 moderately inhibited TNF-α secretion in Caco-2 cells, as shown in Figure 2C. Although sonicated cells of MSMC 57-1 significantly inhibited TNF-α production (* *p* < 0.05), sonicated cells of MSMC 39-1 and MSMC 39-3 did not have a significant effect on TNF-α secretion in the Caco-2 cell line, as shown in Figure 2D.

### 3.2. Effect of Probiotic L. paracasei Strain MSMC39-1 on Body Weight and Stool Consistency in DSS-Induced Colitis Rats

*L*. *paracasei* strain MSMC 39-1 was used in the animal experiment because of its strong TNF-α inhibition property in Caco-2 cells. After 14 days of oral gavage with 3% DSS, weight loss and diarrhea with bloody stool were observed in colitis rats. Although a slight decrease in body weight was observed in the probiotic test group, their stool was normal (Table 2). The body weight and stool consistency of the probiotic control group and the probiotic test group were comparable with the negative control, as shown in Table 2. Therefore, DSS-induced colitis caused weight loss, bloody stool and diarrhea.

### 3.3. Effect of Probiotic L. paracasei Strain MSMC39-1 on Liver Functional Enzyme Activity in DSS-Induced Colitis Rats

**Figure 3:**
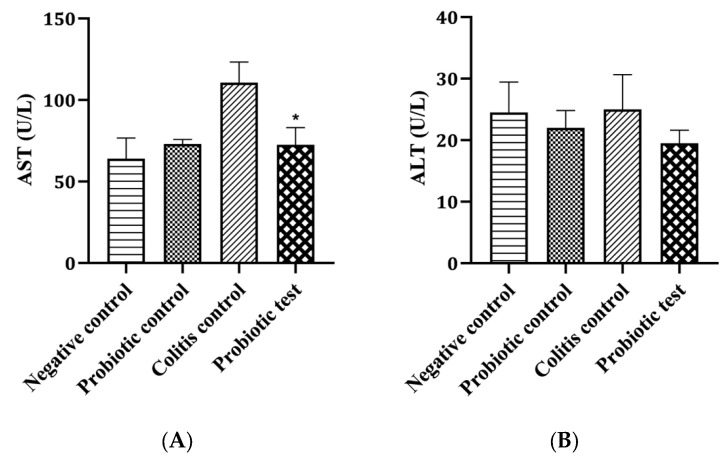
Effect of probiotic *L*. *paracasei* strain MSMC39-1 on liver functional enzyme activity. (**A**) Aspartate aminotransferase (AST) and (**B**) Alanine aminotransferase (ALT) in dextran sodium sulfate-induced colitis rats. Error bar indicated standard deviation. One-way ANOVA was used for statistical analysis and * (*p* < 0.05) indicate significant differences compared to the control (*n* = 6 rats).

The levels of serum asparate aminotransferase (AST) and alanine aminotransferase (ALT) were measured to examine the effect of probiotic *L*. *paracasei* strain MSMC39-1 on the liver function of colitis rats. As shown in Figure 3A, serum AST was elevated in the colitis control group. Feeding *L*. *paracasei* MSMC39-1 to colitis rats, however, significantly decreased AST level when compared with the colitis control group (* *p* < 0.05). Although *L*. *paracasei* strain MSMC39-1 decreased serum ALT level in colitis rats, there was no statistically significant difference between the probiotic-treated colitis rats and the control group (Figure 3B).

### 3.4. Effect of Probiotic L. paracasei Strain MSMC39-1 on TNF-α Production in Colon and Liver Tissues of DSS-Induced Colitis Rats

**Figure 4:**
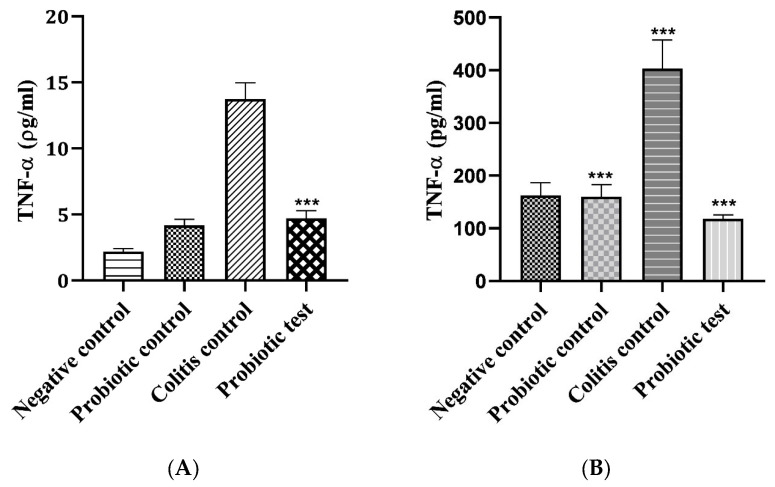
Effect of *L. paracasei* strain MSMC 39-1 on Tumor Necrosis Factor-α (TNF-α) secretion. (**A**) colon tissue and (**B**) liver tissue in colitis rats. Error bars indicate standard deviation. One-way ANOVA was used for statistical analysis and *** (*p* < 0.001) indicate significant differences compared to the control, *n* = 6 rats.

To examine the effect of *L*. *paracasei* strain MSMC39-1 on TNF-α production, an ELISA kit was used to detect the levels of TNF-α in the colon and liver tissues of each rat. Elevations of TNF-α production were observed the in colon and liver tissues of colitis rats (Figure 4A, B). Nevertheless, the levels of TNF-α in these tissues were significantly reduced to similar levels to those of the normal control groups when the probiotic *L*. *paracasei* strain MSMC39-1 was fed to the colitis rats (*** *p* < 0.001 when compared to the colitis control group). Therefore, these data demonstrated that *L*. *paracasei* strain MSMC39-1 treatment can improve colitis by decreasing TNF-α secretion.

### 3.5. Effect of Probiotic L. paracasei Strain MSMC39-1 on Colon and Liver Tissue Histology in DSS-Induced Colitis Rats

**Figure 5:**
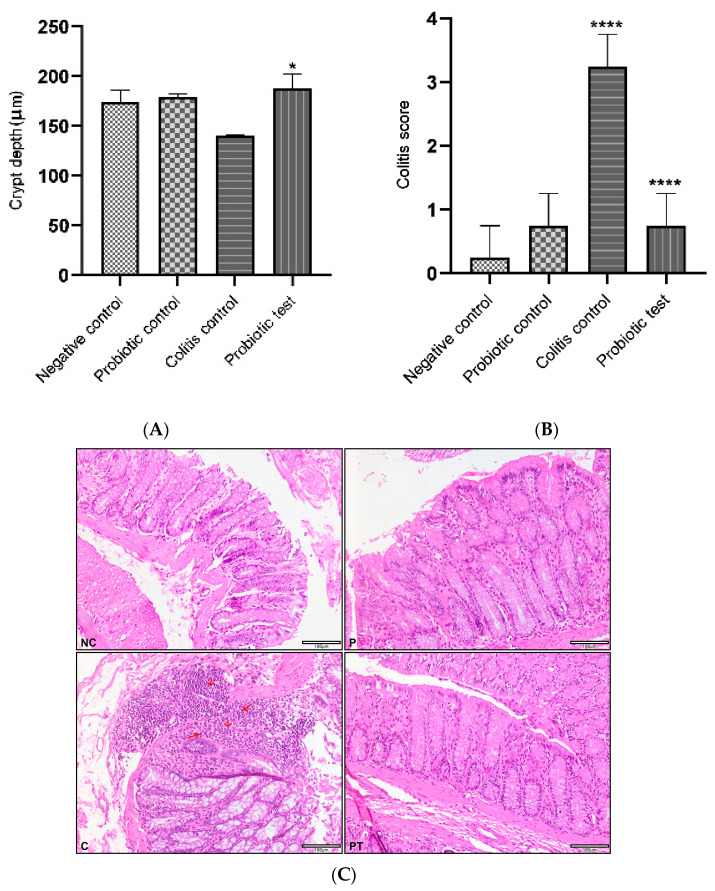
*L*. *paracasei* strain MSMC39-1 treatment on colon in DSS-induced colitis rats. (**A**) Colitis score, (**B**) Crypt depth and (**C**) Colon histology in colitis rat. Normal or negative control (NC) and probiotic control (P) indicated normal colon. Colitis (C) indicated intense inflammatory cells (as indicated by the red arrows), which decreased with *L*. *paracasei* strain MSMC39-1 treatment (PT). Measurement of crypt depth (CD), 200× magnification and scale bar 100 μm. Experiments were performed in duplicate, *n* = 6 rats, negative control. One-way ANOVA was used for statistical analysis. Error bars indicate standard deviations, * (*p* < 0. 05); **** (*p* < 0.0001).

**Figure 6:**
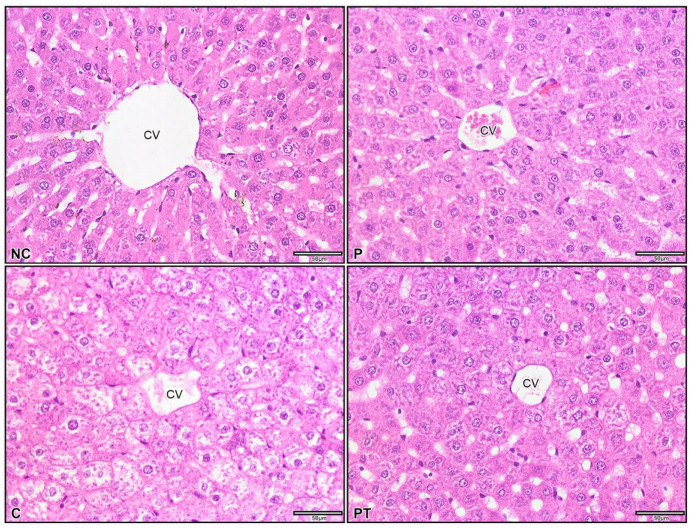
Liver histology of *L*. *paracasei* MSMC39-1 treatment in colitis rats. Normal control (NC) and probiotic control (P) showed normal colon. Colitis (C) indicated intense fat droplets were inside hepatocytes, and this was alleviated by probiotic *L*. *paracasei* strain MSMC39-1 treatment (PT). Central vein (CV), Scale bar 50 μm and 400× magnification.

Hematoxylin and eosin staining were used to examine the effect of probiotic *L*. *paracasei* strain MSMC39-1 on colon and liver histology. Colon tissues from normal control and probiotic control rats showed no inflammation and ulceration with a normal size of colon. The colitis scores of these tissues were 0–1, as shown in Figure 5A–C. In addition, for these rats, crypt depth was decreased, with a crypt abscess and colitis score of 3, as shown in Figure 5A–C. Interestingly, the administration of viable cells of probiotic *L*. *paracasei* strain MSMC39-1 significantly attenuated the colon tissues of colitis rats. As shown in Figure 5A, the number of inflammatory cells in the laminar propria decreased with no sign of ulceration. Moreover, crypt depth was increased and the colitis score was approximately 1 (Figure 5A,B). The crypt depths of normal control, probiotic control and probiotic test groups were similar, as shown in Figure 5B. The infiltration of inflammatory cells in the lamina propria with ulcerations (as indicated by the red arrow) was observed in the colon tissues of colitis rats, as shown in Figure 5C. For liver tissues, modifications of liver architecture was observed in colitis rats. These livers showed areas of inflammation, accumulation of fat droplets and inflammatory cells, swelling of hepatocytes and disarrangement of hepatic cord (Figure 6). Interestingly, feeding with the probiotic *L*. *paracasei* strain MSMC39-1 improved these conditions in colitis rats. There were fewer areas of inflammation and less fat droplet accumulation in the hepatocytes of the liver tissues of these rats. In addition, normal lobular architecture and cell structure were observed in the normal control and probiotic control groups (Figure 6).

### 3.6. Effect of L. paracasei Strain MSMC39-1 on Microbiota Modulation in DSS-Induced Colitis Rats

**Figure 7:**
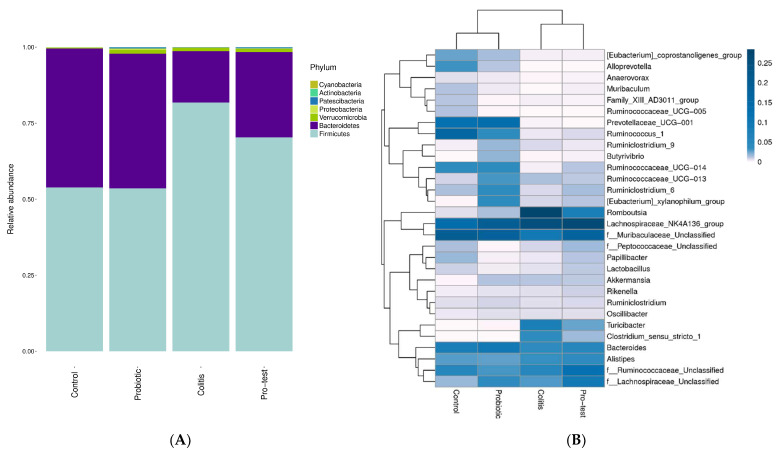

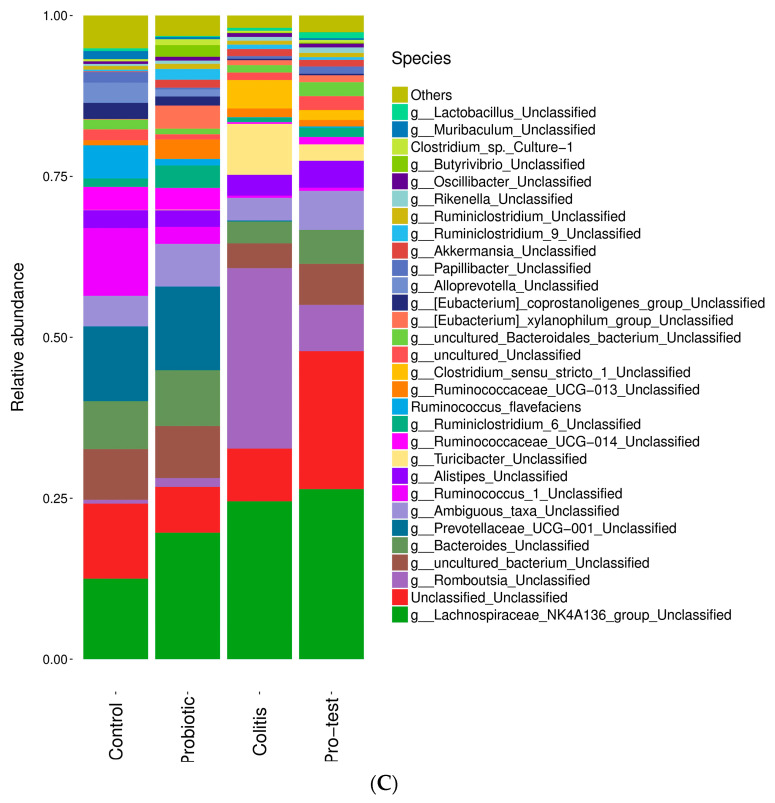
Effect of probiotic *L*. *paracasei* strain MSMC39-1 on microbiota modulation showing (**A**) Phylum, (**B**) Genus and (**C**) Species in colitis rats (*n* = 6 rats).

A next-generation sequencing technique was used to examine the effect of *L*. *paracasei* strain MSMC39-1 on microbiota modulation. As shown in Figure 7A, the number of organisms in the phylum Fermicutes in the feces of the colitis control group was more than that of the probiotic treatment group. A slight decrease in these organisms was observed when viable cells of *L*. *paracasei* strain MSMC39-1 were orally fed to colitis rats. In addition, organisms in the phylum Bacteroidetes were increased in these rats when compared with the colitis control group (Figure 7A). Furthermore, the heat map result showed an increase in the genus *Lactobacillus* spp. in colitis rats treated with *L*. *paracasei* MSMC39-1 when compared with the colitis control rats, as shown in Figure 7B. On the other hand, the number of genus *Clostridium* spp. or pathogenic bacteria was higher in the colitis control group than in the group treated with the probiotic *L*. *paracasei* strain MSMC39-1, as shown in Figure 7C.

## 4. Discussion

In the in vitro study, viable cells and heat-killed cells of probiotics *L*. *paracasei* strain MSMC39-1, *L*. *casei* MSMC39-3 and sonicated cells of *W. confusa* MSMC57-1 suppressed TNF-α secretion in Caco-2 cells. Viable cells of *L*. *paracasei* strain MSMC39-1 were used in colitis rat models due to their strongest suppression effect on TNF-α production in LPS-stimulated Caco-2 cells. Patterns of probiotic antigen such as heat-killed cells and sonicated cells reduced TNF-α in Caco-2 cells because peptidoglycans, lipoteichoic acids or heat labile pili in cell wall components play major roles in immunomodulation [34]. Viable cells of *L*. *paracasei* MSMC39-1 reduced TNF-α via their metabolites and immunomodulation process [35].

Dextran sulfate sodium (DSS) is a chemical reagent used for the induction of ulcerative colitis or colon inflammation. DSS disrupts intestinal barrier function leading to increased leaky gut and antigen and pathogenic bacteria penetration into the lumen. This in turn activates immune cells to secrete pro-inflammatory cytokine (TNF-α and IL-1β), causing intestinal inflammation [36]. Moreover, endotoxins from pathogenic bacteria passing from the leaky gut into the liver via the portal vein bind to toll-like receptor-4 (TLR-4) on hepatocytes and activate Kupffer cells to secrete pro-inflammatory cytokines and stimulate liver inflammation pathogenesis [37]. The symptoms of colitis are body weight loss, bloody diarrhea, ulcers of epithelial cells, infiltration of inflammatory cells, crypt edema, colon shortening and microbiota alteration [38].

In the colitis experiment, the scores of colon inflammation calculated as disease activity indexes (DAI) included body weight, stool and rectal bleeding. The probiotic *L*. *paracasei* strain MSMC39-1 improved DAI scores and the severity of colitis. ALT and AST are liver function enzymes found in hepatocytes that can be used to indicate liver damage [39]. In this study, AST and ALT were high in the serum of the colitis rat control group and decreased in the probiotic strain MSMC39-1 treatment. This result shows that DSS induces colitis and leads to liver injury. This is similar to a recent study, in which *B*. *longum* LC67 and *L*. *plantarum* LC27 significantly reduced ALT and liver injury in mice with 2, 4, 6-trinitrobenzesulfonic acid (TNBS)-induced colitis in a mouse model [40]. The increase in TNF- α production in colon and hepatic tissues of the colitis rat control group was significantly reduced by oral gavage with the probiotic *L*. *paracasei* strain MSMC39-1. Therefore, *L*. *paracasei* strain MSMC39-1 decreased colon and liver inflammation in colitis rats. Colitis rats treated with *L*. *paracasei* strain MSMC39-1 showed less ulceration, reduced colon and liver inflammation and less fat droplets in the hepatocytes. Previously, similar results reported that *B*. *breve* CCFM683 reduced colon inflammation by increasing colon length and reducing TNF-α, IL-1β and IL-6 expression in the colons of DSS-induced colitis mice [41]. A recent study reported that viable and heat-killed cells of *L*. *plantarum* decreased the DAI score of colitis and reduced pro-inflammatory cytokines in the serum and colon of DSS-induced colitis rats [42]. *L*. *casei* LC2W improved symptoms of colitis induced with *E*. *coli* O157:H7 by protecting tight junctions and reducing IL-1β, TNF-α and IL-6 in the colon [43]. *L*. *bulgaricus* and *S*. *thermophilus* in yogurt alleviated the symptoms of colitis in mice and induced IL-2 and IL-4 production in the lymph nodes and spleen, resulting in modulated helper T cells [44]. Similar to previous studies, synbiotic LGG and prebiotic tagatose reduced colitis symptoms in DSS-induced colitis mice [45]. *L*. *fermentum* improved colitis, reduced TNF- α, IL-1β and IL-6 in serum and induced anti-inflammatory cytokine (IL-10) expression in the colons of DSS-induced colitis mice [46]. Furthermore, wheat germ-apple fermented with *L*. *delbrueckii* subsp. *bulgaricus*, *L*. *paracasei*, *L*. *plantarum* subsp. *plantarum*, *L*. *helveticus* and *L*. *plantarum* probiotics mixed with *B*. *infantis*, *L*. *acidophilus*, *E*. *faecalis* and *Bacillus cereus* inhibited pro-inflammatory cytokine production in colon tissues and protected tight junctions in colitis model [47,48,49].

Microbiota modulation was observed in rats with oral administration of viable cells of *L*. *paracasei* strain MSMC39-1. Although the number of phylum Bacterioidetes was more than the phylum Firmicutes in these rats, the heat-map showed an increase in the genus *Lactobacillus* in probiotics-treated rats. An increase in the genus *Clostridium* and a decrease in the genus *Lactobacillus* were observed in colitis rats. Similarly, *L*. *plantarum* Zhang LL, yogurt mixed with *L*. *bulgaricus* and *S*. *thermophilus*, synbiotic of *L*. *rhamnosus* GG and probiotics mixed with *B*. *infantis*, *L*. *acidophilus*, *E*. *faecalis* and *B*. *cereus*, *B*. *breve* CCFM683 increased the genera *Lactobacillus* and *Bifidobacterium* in DSS colitis models [42,44,45,49,50]. Therefore, dysbiosis in the GI tract that is caused by the reduction of microbiota diversity including Fermicutes and Bacterioidetes can be prevented with probiotics [51]. The results of this study revealed the potential of probiotic strains in the prevention of colitis and other inflammation diseases. Further studies may involve the development of various probiotic supplement products, such as juices and protein soups, as well as the testing of conditions for the probiotic production process before the industrial production level.

## 5. Conclusions

In conclusion, the findings indicated that *L*. *paracasei* strain MSMC39-1 inhibited pro-inflammatory cytokine TNF-α secretion in Caco-2 colon carcinoma cells. Orally administered *L*. *paracasei* MSMC39-1 decreased colon inflammation and modulated beneficial microbiota by increasing genus *Lactobacillus* in DSS-induced colitis rats. Further studies should be conducted to confirm the protective roles and other functions of *L*. *paracasei* MSMC39-1 in DSS-induced colitis.

## Figures and Tables

**Table 1:** Colitis score of dextran sulfate sodium-induced colitis rats.

Score	Characteristics
0	no ulcer and inflammation
1	no ulcer and local hyperemia
2	ulcer without hyperemia
3	ulcer and one site only of inflammation
4	two or more sites of ulcer and inflammation
5	ulcer extending more than 2 cm

**Table 2:** Effect of *L*. *paracasei* strain MSMC39-1 on body weight and stool consistency.

	Group			
Parameters	Negative Control	Probiotic Control	Colitis Control	Probiotic Test
Body weight increase (g)	125.00 ± 9.98	122.50 ± 23.91	93.41 ± 6.80	107.40 ± 11.03
Stool consistency	Formed	Formed	Loose, bloody	Formed

## Data Availability

Not applicable.
